# Spontaneous choledochoduodenal fistula with tuberculous duodenal ulceration

**DOI:** 10.1308/003588414X13824511649292

**Published:** 2014-01

**Authors:** VS Karthikeyan, SC Sistla, D Ram, SM Ali, N Rajkumar, G Balasubramaniam, MS Sanker

**Affiliations:** Jawaharlal Institute of Postgraduate Medical Education and Research, Puducherry,India

**Keywords:** Choledochoduodenal fistula, Tuberculosis, Pericholedochal lymph nodes

## Abstract

Spontaneous choledochoduodenal fistulas (CDFs) are rare. The most common aetiology is penetrating duodenal ulcers, observed in 80% of cases. Even in areas where acid peptic disease is common, tuberculosis should be considered as a cause, especially in developing countries like India, where tuberculosis is common. The management of CDF due to acid peptic disease is predominantly surgical while healing of tuberculous CDF has been reported with antitubercular treatment. A preoperative diagnosis of tuberculous CDF by endoscopic biopsy from the duodenal ulcer or image guided fine needle aspiration if abdominal lymph nodes are present can eliminate the need for surgery and achieve a cure with antitubercular treatment. The CDF in this case was due to caseation of periduodenal lymph nodes rupturing into the duodenum and the bile duct.

Spontaneous choledochoduodenal fistulas (CDFs) are rare and the most common aetiology is penetrating duodenal ulcers, observed in 80% of cases.^1^ The other causes include bile duct stones, echinococcal disease, gastric and pancreatic tumours,^2^ and rarely tuberculosis (TB). Although CDF is usually attributed to a duodenal ulcer in areas where acid peptic disease (APD) is endemic, TB should be considered as a common cause in developing countries like India, where it is common. CDF with duodenal ulceration and common bile duct erosion following caseation of periduodenal lymph nodes in TB has been reported rarely in the literature. We therefore report this case for its rarity and to review the management of tuberculous CDF.

## Case history

**Figure 1:**
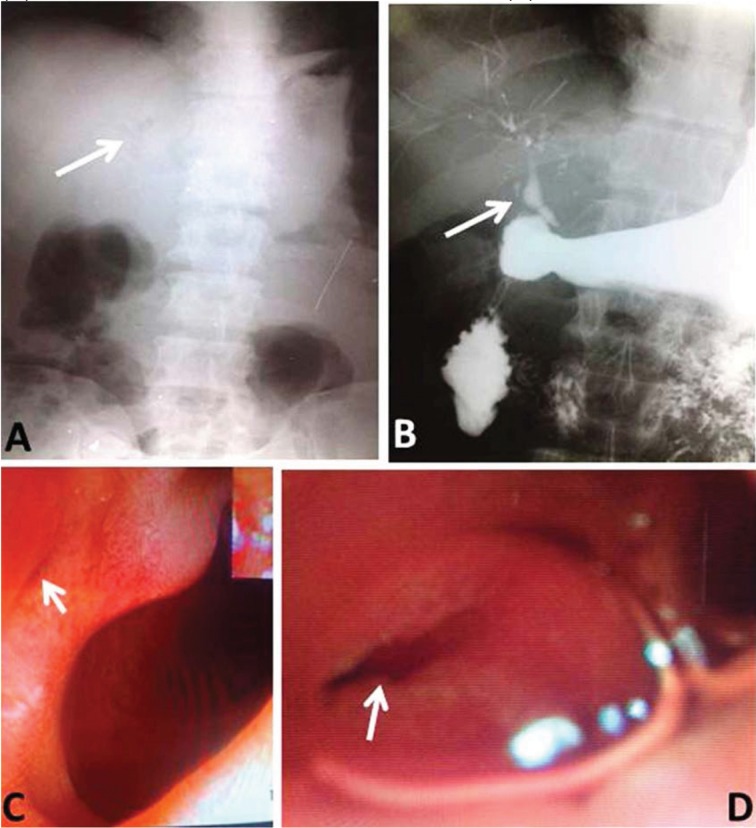
Choledochoduodenal fistula: plain x-ray of the abdomen showing pneumobila (A), water soluble contrast study showing contrast leak from duodenum into biliary tract (B), oesophagogastroduodenoscopy showing duodenal lumen and choledochoduodenal fistula (C), and closer view of choledochoduodenal fistula (D)

A 53-year-old man presented with a 3-year history of recurrent abdominal pain localised to the upper abdomen. The pain was moderate in intensity and occurred on an empty stomach. It was relieved with food intake. There was no associated nausea or vomiting. There was no history of associated co-morbidities. Since the symptoms were suggestive of APD, an oesophagogastroduodenoscopy was performed, which found an ulcer in the first part of the duodenum (Fig 1). A diagnosis of a duodenal ulcer was made and he was given two-week course of *Helicobacter pylori* eradication therapy with omeprazole, clarithromycin and amoxicillin.

As there was no improvement in symptoms following treatment, ultrasonography of the abdomen was performed to look for other causes. This revealed pneumobilia with a 2.2cm × 1.9cm well defined hypoechoic lesion close to the proximal body of the pancreas, suggestive of a peripancreatic lymph node, and a 1cm × 0.9cm non-shadowing echogenic focus in the gallbladder, suggestive of sludge. Pneumobilia was also demonstrated on plain x-ray of the abdomen (Fig 1). An upper gastrointestinal contrast study was carried out to diagnose the aetiology, which revealed a CDF. A repeat oesophagogastroduodenoscopy was performed and the fistula was clearly made out (Fig 1).

**Figure 2:**
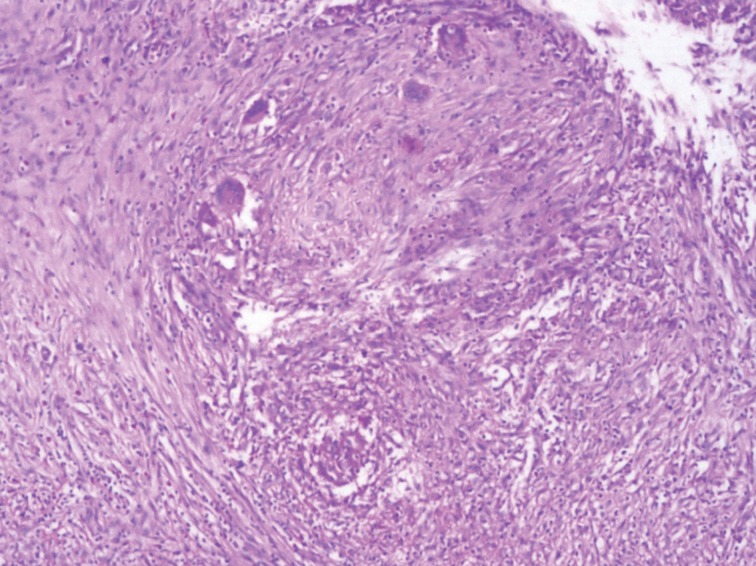
Histopathology of peritoneal nodule showing caseating granulomas

Based on these findings, a provisional diagnosis of chronic duodenal ulcer with CDF with chronic cholecystitis was considered and the patient underwent open cholecystectomy and truncal vagotomy with gastrojejunostomy. Intraoperatively, a nodule with a 1cm × 1.5cm fistulous opening was found in the duodenum, with multiple peritoneal deposits. The possibility of TB was considered. On histopathology, the gallbladder showed chronic cholecystitis with intestinal metaplasia and the peritoneal nodules revealed caseation with epitheloid granulomas suggestive of TB (Fig 2).

Postoperatively, the patient was investigated for other foci of TB and retropositive status, both of which were negative. He was put on Revised National Tuberculosis Control Program category I antitubercular therapy with isoniazid, rifampicin, ethambutol and pyrazinamide for two months, followed by isoniazid and rifampicin for four months. He has been on regular follow-up for the past three years and remains asymptomatic.

## Discussion

Bilioenteric fistulas seldom produce clinical symptoms^3^ and are diagnosed incidentally in most cases. CDF is a relatively rare form of biliary-intestinal fistula and it contributes to 3.5–20% of all internal biliary fistulas.^2,4^ Eighty per cent of these CDFs are secondary to penetrating duodenal ulcers.^1^ Stones in the bile duct, echinococcal disease and, rarely, tumours of the stomach and pancreas are the other known causes of CDF.^2^ TB causing duodenal ulceration and common bile duct erosion due to caseation of periduodenal lymph nodes is quite rare.

Gastroduodenal TB is rare even in patients with pulmonary TB and on autopsy, the incidence is 0.5%.^5^ Duodenal involvement contributes to only 2.5% of TB enteritis.^4^ This exceedingly rare incidence is attributable to the high acidity and paucity of lymphoid tissue in the duodenum as well as rapid transit of food in the gastroduodenal area,^6^ which is hostile to the tubercle bacilli. There is usually extensive lymph node involvement in the surrounding regions.^7^

Although a duodenal ulcer is the most common cause of CDF in areas endemic for APD, TB should be considered as another potentially common cause in developing countries. Since duodenal ulcers are usually considered to be due to APD, it is not customary to perform a biopsy to establish the diagnosis. However, when a patient presents with CDF, it may be beneficial to establish the aetiology preoperatively (ie whether it is a penetrating duodenal ulcer secondary to APD or whether it is TB). This is important because the management of these two conditions is entirely different. Operative management is the treatment of choice in duodenal ulcers caused by APD whereas medical management is highly effective in tuberculous aetiology and antitubercular treatment has been shown to cause healing of CDF.^2^

The diagnosis of TB as an aetiology for duodenal ulcer is difficult and is often confused with APD.^5^ Diagnosis of TB centres around the demonstration of acid fast bacilli or caseous granulomas on biopsy. However, these biopsies are positive only in a third of cases.^5^ On most occasions, the diagnosis is made only after exploring the abdomen.^5^ Preoperative ultrasonography of the abdomen in CDF cases can therefore be beneficial if lymph nodes are visualised and image guided fine needle aspiration from these nodes may help in establishing the diagnosis of TB preoperatively.

The treatment of CDF in this situation is directed primarily at relieving the duodenal ulcer disease and diversion of the bile flow. When surgery is recommended, the operation of choice is a truncal vagotomy with an antrectomy or a gastrojejunostomy, leaving the fistula intact.^8^ Surgical procedures such as cholecystectomy, common bile duct exploration and bilioenteric reconstruction are reserved for the rare event of a biliary stricture. In our case, a truncal vagotomy with a gastrojejunostomy was performed because the preoperative diagnosis was a duodenal ulcer following APD with CDF. Multidrug antitubercular treatment regimens can cure TB and have been shown to result in closure of the fistulous tract.^2^

## Conclusions

Although TB is a rare cause of CDF, it should always be considered in developing countries like India, where TB is common. If the diagnosis of TB can be established preoperatively, by means of endoscopic biopsy from the duodenal ulcer or image guided fine needle aspiration if abdominal lymph nodes are seen, antitubercular treatment can be curative, eliminating the need for surgery.

## References

[1] Hoppenstein JM, Medoza CB, Watne AL. Choledochoduodenal fistula due to perforating duodenal ulcer disease. *Ann Surg* 1971; : 145–147. 10.1097/00000658-197101000-00022 PMC1397128 5543545

[2] Chaudhary A, Bhan A, Malik N *et al.* Choledocho-duodenal fistula due to tuberculosis. *Indian J Gastroenterol* 1989; : 293–294. 2599567

[3] Michowitz M, Farago C, Lazarovici I, Solowiejczyk M. Choledochoduodenal fistula: a rare complication of duodenal ulcer. *Am J Gastroenterol* 1984; : 416–420. 6720662

[4] Miyamoto S, Furuse J, Maru Y *et al.* Duodenal tuberculosis with a choledochoduodenal fistula. *J Gastroenterol Hepatol* 2001; : 235–238. 11207910 10.1046/j.1440-1746.2001.02332.x

[5] Lamberty G, Pappalardo E, Dresse D, Denoël A. Primary duodenal tuberculosis: a case report. *Acta Chir Belg* 2008; : 590–591. 19051473 10.1080/00015458.2008.11680292

[6] Rao YG, Pande GK, Sahni P, Chattopadhyay TK. Gastroduodenal tuberculosis management guidelines, based on a large experience and a review of the literature. *Can J Surg* 2004; : 364–368. 15540690 PMC3211943

[7] Agrawal S, Shetty SV, Bakshi G. Primary hypertrophic tuberculosis of the pyloroduodenal area: report of 2 cases. *J Postgrad Med* 1999; : 10–12. 10734324

[8] H’ng MW, Yim HB. Spontaneous choledochoduodenal fistula secondary to long-standing ulcer disease. *Singapore Med J* 2003; : 205–207. 12952034

